# Expansion of Smartwatch Touch Interface from Touchscreen to Around Device Interface Using Infrared Line Image Sensors

**DOI:** 10.3390/s150716642

**Published:** 2015-07-09

**Authors:** Soo-Chul Lim, Jungsoon Shin, Seung-Chan Kim, Joonah Park

**Affiliations:** Device & System Research Center, Samsung Advanced Institute of Technology, 130 Samsung-ro, Yeongtong-gu, Suwon-si, Gyeonggi-do 443-803, Korea; E-Mails: soochul.lim@samsung.com (S.-C.L.); jsoon.shin@samsung.com (J.S.); sc12.kim@samsung.com (S.-C.K.)

**Keywords:** smartwatch touch interface, infrared image sensor, around device interface

## Abstract

Touchscreen interaction has become a fundamental means of controlling mobile phones and smartwatches. However, the small form factor of a smartwatch limits the available interactive surface area. To overcome this limitation, we propose the expansion of the touch region of the screen to the back of the user’s hand. We developed a touch module for sensing the touched finger position on the back of the hand using infrared (IR) line image sensors, based on the calibrated IR intensity and the maximum intensity region of an IR array. For complete touch-sensing solution, a gyroscope installed in the smartwatch is used to read the wrist gestures. The gyroscope incorporates a dynamic time warping gesture recognition algorithm for eliminating unintended touch inputs during the free motion of the wrist while wearing the smartwatch. The prototype of the developed sensing module was implemented in a commercial smartwatch, and it was confirmed that the sensed positional information of the finger when it was used to touch the back of the hand could be used to control the smartwatch graphical user interface. Our system not only affords a novel experience for smartwatch users, but also provides a basis for developing other useful interfaces.

## 1. Introduction

People are increasingly relying on mobile devices in their daily life. Recently, smartwatches were proposed to provide users with instant access to digital activity. The miniaturization of computing devices has enabled smartwatches to perform almost all the functions of smartphones, including the making of telephone calls, and they can also be operated as stand-alone devices. Smartwatches have two strong advantages over smartphones, namely, the mounting location, and the continuous contact with the skin [1]. They have light weight, and mounting on the wrist affords the user immediate access to messages, notifications, and other digital data. Their continuous contact with the wrist also enables their being used for tracking purposes and to obtain health information such as heart rate and oxygen saturation. These advantages have prompted many manufacturers to engage in the production of smartwatch products. The development of smartwatch computing hardware that are comparable with those of mobile phones was commenced a few years ago, and sufficient computing power has now been achieved that allows smartwatches to be used as platforms for several mobile applications. However, smartwatches are limited by the small available interactive surface area and space for accommodating physical buttons for user interaction. This compactness of smartwatches and wrist-wearable devices is the reason why most of them have small or no touchscreens. When used, the small size of the touchscreen limits its functionality in terms of controlling the graphical user interface with a finger, and the visual feedback to the user is also obscured by the finger [2]. While the expansion of the input methods would significantly increase the usability of smartwatches, which are becoming increasingly small, the touchscreen is the only way to control a current smartwatch.

Many interfaces for smartwatches require using the fingers to touch somewhere on the device. Finger gestures have become familiar and acceptable methods for general interaction with mobile devices and they afford users intuitive manipulation techniques. We therefore propose a method for interacting with a smartwatch by touching the back of the hand instead of the smartwatch screen. By using a finger position sensor around the device to track the position of the finger on the back of the hand, the interaction area is moved away from the screen of the smartwatch. In this paper, we describe a prototype device with compact IR line array sensors embedded along one side of the smartwatch, capable of detecting the position and swipe direction of a finger gesture on the back of the hand.

This rest of this paper is organized as follows: in Section 2, we describe relevant previous studies on wrist-wearable interfaces and finger-motion interfaces. In Section 3, we describe the configuration of the hardware of our proposed system, how the finger position is measured, and the performance evaluation of the system. Section 4 is concerned with the implementation of an around device interface module suitable for smartwatches. We finally draw our conclusions and note the scope of further study in Section 5.

## 2. Background

Around device interfaces (ADIs) are increasingly being investigated as efficient interaction means of expanding the interaction space of small devices [3,4,5]. ADIs are useful for controlling small wearable mobile devices such as mobile phones, wristwatches, and head-mounted displays. Hally [6] showed that the wrist and forearm are “useful” and “ease to use” points for the on-the-body placement of the interface of a wearable system. This has led to the proposal of a number of alternative solutions for expanding the input interaction surface of such systems. Harrison [7] used mechanical vibration propagation through the body as an input interface. Skin buttons [8] that use infrared (IR) proximity sensors as click icons for interaction with smartwatches have also been proposed. HoverFlow [3] is a technology that uses IR sensors to implement hovering gesture recognition above the screen of mobile devices. Nakasuma [9] measured the finger touch position on the back of the hand using an IR reflector. SideSight [10] also uses IR distance sensors to implement multi-touch input in areas on the sides of a device.

To capture more detailed gestures, poses, and commands, hand gesture/pose recognition is widely employed. The technology utilizes an image sensor [5,10,11,12] or an RGB-D camera [13,14,15]. Digits [16] is a wrist-worn sensor that captures the full 3D pose of the user’s hand by background subtraction using an IR laser and camera. However, these image-based recognition methods require the mobile device or smartwatch to execute many computations to interpret the gesture or pose of the hand. Unfortunately, the small form factor of the devices limits the hardware resources that can be accommodated. In the implementation of ADI in a mobile device or smartwatch, many sensors are used to determine the touched position based on a few calculations. Liang [17] proposed a method for interacting with smartwatches by detecting the finger-tapped position on the arm based on the flight time of an air-propagated ultrasound signal. Point Upon Body [18] is another method that has been proposed for localizing the finger position on the forearm using oblique ultrasonic rangefinders. The temporal patterns of gestures such as tapping and swiping have also been used as the basis for sending different interaction commands to mobile devices. Ketabdar [19] designed a means of interacting with the 3D space around a device using magnetic fields. The movement of a magnet attached to the finger affects the magnetic field sensed by a compass sensor integrated in the device. Jing also developed an accelerometer-based ring-shaped device that functions as a human-machine interface for detecting finger gestures, and performs other purposes such as appliance control [20]. Kim developed a system for interpreting a pinch-to-zoom gesture using surface electromyography signals and the distance between the thumb and the index finger [21]. The present study built upon the findings of previous studies that demonstrated the benefits of wearable devices being mounted on the wrist [3,22,23] for easy eyes-free and always-available interaction.

## 3. Around Device Interface

We developed an around device interface for interaction with a smartwatch by sensing the finger gesture and its touch position on the back of the hand using sensing the finger reflected IR intensity and its position.

### 3.1. Around Device Interface System

**Figure 1:**
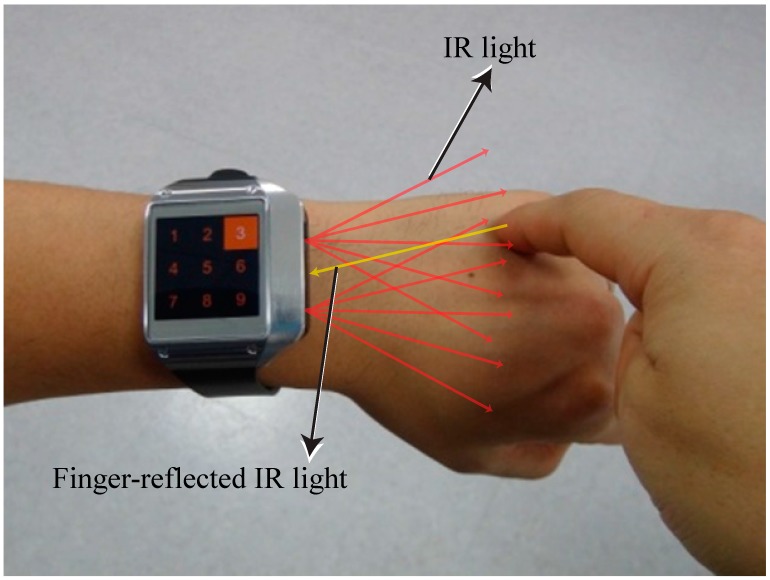
Interaction with a finger gesture by touching the back of the hand.

**Figure 2:**
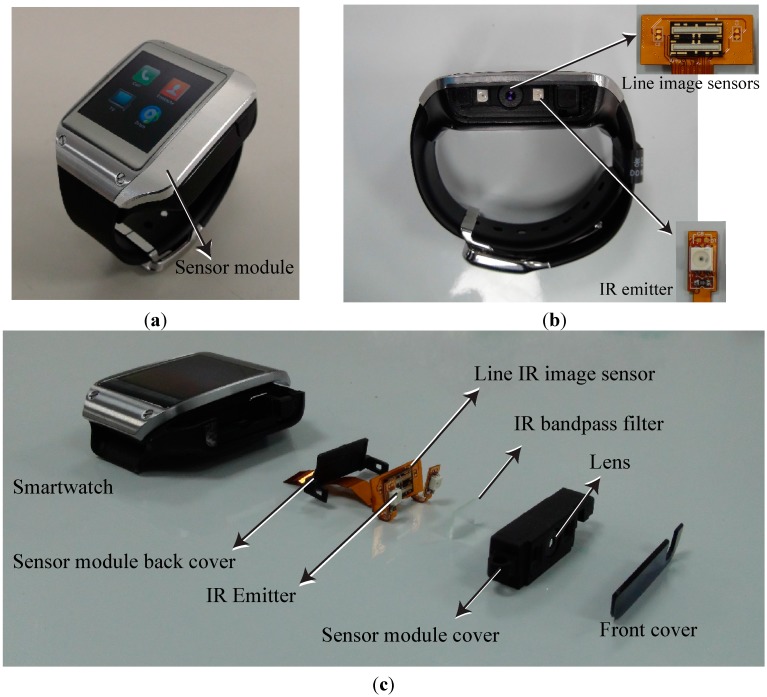
(**a**) Prototype smartwatch; (**b**) side view of prototype smartwatch showing the line image sensor and IR emitter; (**c**) components of the prototype sensor module, including IR line image sensor and emitter.

Figure 1 illustrates the interaction method, wherein a finger is used to touch the back of the hand. Two IR emitters and sensors located on one side of the smartwatch are used to sense the position touched by the finger. We integrated an IR emitter and optical sensor in the side of a Samsung Galaxy Gear smartwatch. Figure 2 shows the prototype smartwatch and the module used to sense the finger-touched position on the back of the hand. Sensors with lenses were placed in the middle of one side of the smartwatch, and two emitters were placed on the right and left of the lens, respectively, to cover the back of the hand (Figure 2b). Figure 2c shows the components of the prototype sensor module. Two commercially available IR line image sensors and a 128 × 1 array photo detector (TSL 1401, AMS, Unterpremstaetten, Austria) are used to measure the position of the finger and its gesture by detecting the light emitted from two IR light-emitting diodes (LED, SFH-4250, OSRAM, Munich, Germany) and that reflected by the finger. We used wide-angle IR LEDs to cover a large interaction area within a short distance. The short-distance interaction reduces the LED power consumption compared to typical middle- or long-range time-of-flight measurement devices. To eliminate external light, we used an IR band-pass filter with a range of 850 ± 10 nm. An aspheric lens was installed and focused on the object to widen the viewing angle within a short distance. A gyro sensor embedded in the Samsung Galaxy Gear is used.

We measured the finger position on the back of the hand by sensing the intensity of the IR light reflected by the finger. When the user’s finger touched the back of the hand, the IR intensity was highest at the finger position because it was nearest to the emitter. From the location of the peak pixel intensity relative to the IR line image sensor in each frame, we could determine the two-dimensional coordinates of the finger on the back of the hand. In addition, to detect the finger position before a touch motion, it was necessary to locate it horizontally using two line sensors. The IR line sensors at the bottom are capable of sensing in a plane inclined at more than 10° relative to the center of the lens.

### 3.2. Finger Position Measurement and Performance Evaluation

**Figure 3:**
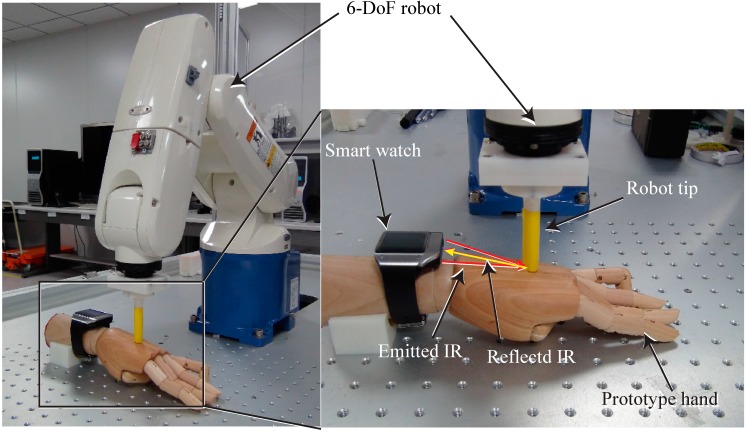
6-DoF robot system for measuring the IR intensity and peak position in the developed smartwatch interface.

**Figure 4:**
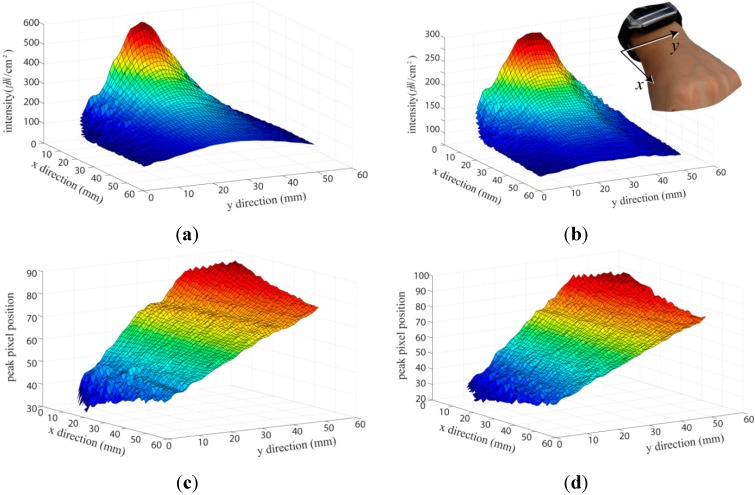
Measurement results of (**a**) IR intensity (upper sensor), (**b**) IR intensity (lower sensor), (**c**) peak pixel position (upper sensor), and (**d**) peak pixel position (lower sensor).

To measure the peak position and the IR intensity at each position on the back of the hand, we used a six-degree-of-freedom (6-DoF) industrial robot (VP-6242G, Denso, Nagoya, Japan). The robot can be controlled with a resolution of 1 mm and the peak position and IR intensity could therefore be measured at each exact position on the back of the hand. The measurement setup is shown in Figure 3. The developed smartwatch was placed on a prototype hand, and the peak positions and IR intensities were measured when the robot used a tip with a diameter of 12 mm to touch the back of the mockup hand. The tip was moved over the back of the hand and the peak position and IR intensity at each position were measured with a resolution of 1 mm. Figure 4 shows the raw data of the peak position and maximum IR intensity of the signal from the IR line image sensor. Figure 4a,b shows the maximum IR intensities of the upper and lower sensors for each robot tip position. The line sensor receives the light reflected by the finger, and the acquired data has nonlinear characteristics. In Figure 4a,b the intensity at the center in the *y* direction is higher than those in the side areas at the same distance from the smartwatch (same tip position in the *x* direction) because the viewing angles of the two IR emitters overlapped. Figure 4c,d show the pixel positions with the maximum IR intensity of the signal from the IR line sensor for each robot tip position. The pixel position of the maximum IR intensity for a given *x* position increased with increasing *y* position on the back of the hand. These linear characteristics can be used to determine the *y* position touched by the finger.

Additionally, average power consumption of the module was measured during the robot interacts every second with finger for 10 m with screen off. The power consumption of developed module was measured by inserting a sense resistor between the power supply and the developed system. The module can affect overall power consumption by up to 281.2 mW.

**Figure 5:**
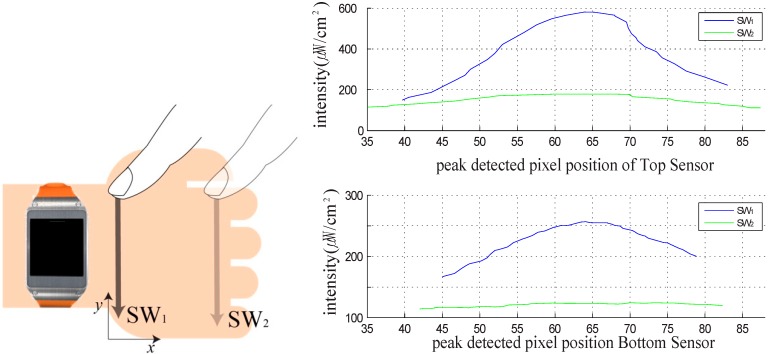
Calibration method and sample results of the relationship between the IR intensity and the pixel position.

Because of the size and color differences among human hands, initial calibration of the finger position is necessary. The calibration method is illustrated in Figure 5. During each of the swipe motions (SW_1_ and SW_2_) at each end of the back of the hand, the maximum peak intensity of each pixel (M_upper sw1_, M_bottom sw1_, M_upper sw2_, and M_bottom sw2_) of the two IR line sensors (upper and lower sensors) for each finger position is measured. By comparing the acquired data with that in Figure 4, the internally saved pre-measured robot-tip position data is remapped. The user can thus use the final calculated *x*-*y* positions on the back of the hand to control the smartwatch.

To evaluate the performance of our developed touch interface, we controlled a robot tip on the back of a mockup hand and implemented a click motion at each position. The robot tip position was selected and moved randomly over the back of the hand, and the peak intensity and pixel location of the two IR line image sensors were measured after the robot motion stopped. The test comprised 50 repetitions of the motion for each click position with a resolution of 1 mm. From the measurement data, the maximum intensity error (*x* direction) was determined to be 11.7%, and the maximum pixel error (*y* direction) was 8.2%. From the measured error, the *x* and *y* directional resolutions were determined to be 7.02 and 4.32 mm, respectively.

### 3.3. Finger Touch Recognition

**Figure 6:**
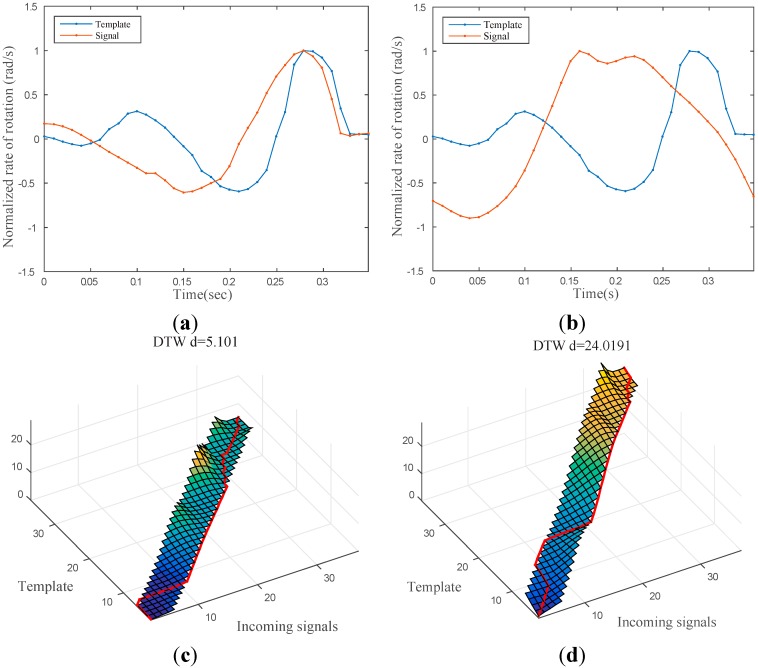
(**a**) Sample time signal while touching the back of the hand; (**b**) sample time signal while rotating the wrist (no touch); **(c**,**d)** DTW cost matrices of samples (a) and (b). The bottom-right corners of (c) and (d) show the final DTW distances of the two signals. It should be noted that (a) is more similar to the template with respect to the DTW distances.

Because the smartwatch is worn on the wrist where a variety of motions frequently take place, the system may produce unwanted identification results. To determine whether a user is intentionally touching the back of his/her hand to interact with the watch, it is necessary to correctly detect the user’s touch status. To accomplish this, we captured the inertial motion signals from the gyroscopic sensor embedded in the smartwatch every 15 ms and measured the time-series similarity between a template signal and the incoming signal using a dynamic time warping (DTW) algorithm, which is robust for measuring time series similarity [24]. A temporal sequence of the *y*-axis gyroscopic data was stored as the template signal and compared with the incoming gyroscope data during the runtime. To achieve efficient operation of the algorithm on wearable devices, we employed a Sakoe-Chiba Band constraint [25] with a width of 10% of the template signal length. Figure 6 shows two examples of matched and unmatched incoming signals, respectively.

## 4. Implementation of Smartwatch Interface with Around Touch

Based on the developed sensor module and around touch interface for a wearable device on the back of the hand, we developed a prototype Android graphical user interface and related applications, which were implemented on the prototype Galaxy Gear. By sensing the finger position using two linear IR sensors and determining the action of the finger through the gyroscope in the smartwatch, the click position and swipe gestures could be read via the watch interface.

**Figure 7:**
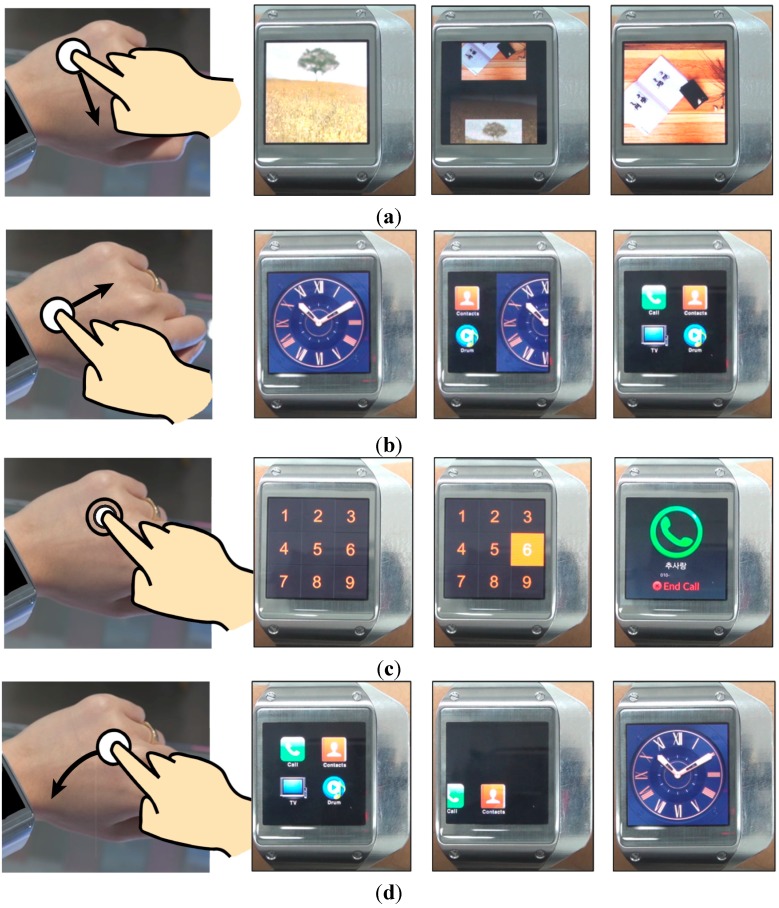
Practical user interface and example application of the Android smartwatch with the developed sensor module: (**a**) rightward swipe gesture to change the mode from the watch mode to the menu of the smartwatch; (**b**) downward swipe gesture to scroll to the next image in the photo gallery application of the smartwatch; (**c**) click gesture to execute a speed dial; (**d**) leftward-downward swipe gesture to operate the “back” button.

Figure 7 shows an example of a practical user interface and application for the Android smartwatch. The screenshots show how each gesture of the user is followed. The smartwatch user interface and applications could be controlled by the developed touch interface. The user interaction consists of swipe and click gestures, which can be used to invoke a variety of functions of the developed smartwatch. The touch boundary line of the x-y position is decided by dividing the area with the amount of the graphical user interface at the calibrated pixel position and intensity, as shown in Figure 4 and Figure 5. The chosen position can be mapped to the position of the graphic user interface element during the user’s click motion. The rightward/leftward swipe gesture is assigned to scroll the menu horizontally, and the upward/downward swipe gesture is assigned to scroll vertically through the photo gallery, telephone directory, *etc.* For example, if the user desires to scroll through the gallery to a certain picture, the finger is used to touch the back of the hand and a downward swipe gesture is executed. The gesture sequentially scrolls to the next picture in the gallery (Figure 7a). A rightward swipe gesture is used to change the mode from the watch mode to the smartwatch menu (Figure 7b). A click gesture on the back of the hand is used to select from the menu or speed dial list, similarly to the operation of an ordinary smartphone. Each area on the back of the hand is mapped to the virtual *x-y* coordinates. Figure 7c shows an example of a click gesture for speed dialing. A leftward-downward swipe gesture anywhere in the user interface is used to operate the “back” button (Figure 7d).

## 5. Conclusions

This paper proposes a new method for interacting with smartwatches using a touch interface on the back of the hand. A finger touch on the back of the hand is recognized by two IR linear array sensors and two IR emitters. For more reliable detection of touch and swipe motions, a gyroscopic sensor in the smartwatch is used to measure the temporal signal with the aid of a DTW algorithm, and the touch status is determined by comparing the signal with a pre-sensed touch signal. The newly developed interface module can be compactly integrated in a smartwatch and operated by simple touch and swipe motions. Further study is ongoing to simplify the required calibration and increase the recognition resolution by a stereo sensor.

## Supplementary Materials

Supplementary materials can be accessed at: http://www.mdpi.com/1424-8220/15/7/16642/s1.
